# Predictive value of clinical risk factors for bladder dysfunction in Syrian patients with type 2 diabetes mellitus

**DOI:** 10.1038/s41598-024-57050-w

**Published:** 2024-03-26

**Authors:** Younes A. Khadour, Bashar M. Ebrahem, Weaam ALHATEM, Engo Ovone Yanne, Fater A. Khadour

**Affiliations:** 1 Department of Physical Therapy, Cairo University, Cairo, 11835 Egypt; 2 Department of Rehabilitation, Faculty of Medicine, Al Baath University, Homs, Syria; 3 Department of Physical Therapy, Health Science Faculty, Al-Baath University, Homs, Syria; 4 Department of Sport Education, Neijiang Normal University, Sichuan, 641004 China; 5 Department of Urology, Tongji Hospital, Tongji Medical College, Huazhong University of Science and Technology, Wuhan, 430030 China; 6 Department of Rehabilitation, Tongji Hospital, Tongji Medical College, Huazhong University of Science and Technology, 1095#, Jie-Fang Avenue, Qiaokou District, Wuhan, 430030 Hubei China

**Keywords:** Bladder dysfunction, Diabetes mellitus, Prediction model, Predictive value, Bladder, Kidney diseases, Epidemiology, Outcomes research, Risk factors, Urology

## Abstract

Diabetes mellitus (DM) is a prevalent disorder that affects the endocrine and metabolic systems. Among the various complications associated with DM, diabetic bladder dysfunction (DBD) is the most frequently occurring genitourinary complication. The presence of DBD can lead to complications that affect the upper urinary tract, significantly impacting the quality of life for individuals with DM. Therefore, it is crucial to identify early risk factors for DBD and predict its onset. Given the absence of studies involving bladder dysfunction in patients with type 2 diabetes mellitus (T2DM) in Syria, this study aims to examine the risk factors associated with bladder dysfunction in T2DM patients and develop a predictive model to identify DBD early. Patients diagnosed with T2DM were enrolled in six endocrinology centers spread across four Syrian provinces between January 2018 and December 2023. Factors that showed an association with DBD in the bivariate analysis, with a significance level of *p* < 0.05, were included in a multiple logistic regression analysis. The logistic regression analysis was used to identify independent risk factors and develop a prediction model. The receiver operating characteristic (ROC) curve was used to assess the predictive performance of the identified risk factors and the prediction model for DBD. One hundred and eighty-four patients were included in this study, and they were divided into the DBD group (*n* = 88) and the non‐DBD group (*n* = 96). Seven variables showed significance in the bivariate analysis. Furthermore, the multiple logistic regression analysis revealed that age (OR [95% CI]: 0.981 [0.614 − 1.337]), *p* < 0.007; diabetic peripheral neuropathy (DPN) (OR [95% CI]: 1.421 [1.027 − 3.308]), *p* = 0.03; glycated hemoglobin (HbA1c) (OR [95% CI]: 0.942 [0.821 − 1.141]), *p* = 0.042; and percentage of monocyte (Mono%) (OR [95% CI]: 1.109 [0.812 − 1.258]), *p* = 0.031 were independent risk factors for DBD. Analysis of the ROC curve revealed that the area under the curve (AUC) for age, DPN, HbA1c, and Mono were 0.703, 0.541, 0.613, and 0.836, respectively. Age, DPN, HbA1c, and Mono% were risk factors for DBD. The prediction model constructed based on the four risk factors had a good predictive value for predicting the occurrence of DBD.

## Introduction

Diabetes mellitus (DM) is a prevalent endocrine and metabolic condition, encompassing both type 1 diabetes (T1DM) and type 2 diabetes (T2DM). The International Diabetes Federation reported that there were 536.6 million adults with diabetes worldwide in 2021, and this number is projected to increase to 783.2 million by 2045^1^. T1DM affects approximately 5–10% of individuals, while T2DM affects 90%–95%^2^. Diabetes can give rise to various complications, including nephropathy and peripheral neuropathy. Among these, diabetic bladder dysfunction (DBD) is the most common genitourinary consequence associated with diabetes^3^. It is estimated that 25%-90% of individuals with diabetes will experience varying degrees of DBD^4^. DBD is characterized by increased bladder capacity, decreased detrusor contractility, and diminished bladder sensation^5^. The occurrence of this condition can lead to complications such as pyelonephritis, ureteral dilatation, and recurrent urinary tract infections (UTIs), significantly impacting patients' quality of life^6,7^. Therefore, early identification of risk factors for DBD and prediction of its development are crucial. However, previous studies have produced conflicting results regarding the risk factors for DBD, likely due to variations in patient selection and definitions of DBD. Age, body mass index (BMI), diabetic peripheral neuropathy (DPN), and depression are recognized risk factors for lower urinary tract symptoms in individuals with diabetes^8–10^.

Additionally, the Diabetes Prevention Program Outcomes Study revealed an association between lifestyle changes, such as weight gain and reduced physical exercise, and lower urinary tract symptoms, including urinary incontinence^11^. Although several factors influence DBD, no practical, accurate, and easy-to-use evaluation tool is currently available for predicting its occurrence. Consequently, we conducted a retrospective study to collect comprehensive clinical data and laboratory test results (including renal function tests and regular blood tests), explore the relevant risk factors of DBD, and construct a prediction model for the early prediction of DBD.

## Methods

### Study population

This retrospective case–control study was conducted at six endocrinology centers in four Syrian provinces: Damascus, Homs, Hama, and Latakia. Two centers were selected from each province. The study included patients with type 2 diabetes mellitus (T2DM) who had visited these centers between January 2018 and December 2023. The diagnosis of type 2 diabetes mellitus was based on WHO guidelines and similar previous articles and was confirmed by a physician in the endocrinology department of our center. Patients with fasting plasma glucose ≥ 7 mmol/L or 2‐hour postprandial glucose ≥ 11.1 mmol/L were diagnosed as diabetic^12–15^.

In this study, the DBD group was defined as patients with T2DM: (1) with one or more of the symptoms of the lower urinary tract (urinary frequency, urgency, increased nocturia, dysuria, incontinence, and urinary retention); (2) bladder residual urine volume was ≥ 50 mL as determined by B ultrasound^16^. The non‐DBD group was defined as patients with T2DM: (1) with or without the symptoms of the lower urinary tract; (2) bladder residual urine volume of < 50 mL.

Patients with T2DM had to have their bladder residual urine volume measured using B-ultrasound to participate in the trial. The exclusion criteria: (1) patients with neuropathy caused by conditions other than diabetes mellitus (such as spinal injury, Parkinson, and stroke); (2) patients with recurring urinary tract infections; (3) patients with prostate diseases (such as benign prostatic hyperplasia, prostate cancer, and prostate surgery); and (4) patients with pelvic disorders (such as pelvic organ prolapse and pelvic surgery).

The study received approval from the Ethical Committee of the Al Baath University Institutional Review Board Consent Letter as indicated by the Consent Letter–IRB 2,023,205-S.

### Collection of data

The medical information used in this study was taken from the patients' electronic medical files as follows: (1) demographic data including body mass index (BMI), duration of T2DM, gender, treatment, ankle reflexes, vibration perception and age; (2) comorbidities including diabetic peripheral vascular disease, coronary heart disease, diabetic nephropathy, hypertension, history of stroke, Hyperlipaemia, and diabetic peripheral neuropathy (DPN); (3) laboratory examination including glycated hemoglobin (HbA1c), urinary microalbuminuria (mALB), triglyceride (TG), total cholesterol (TC), high‐density lipoprotein cholesterol (HDL‐C), low‐density lipoprotein‐cholesterol (LDL‐C), Urea, serum creatinine (SCr), uric acid (UA), red blood cell count (RBC), white blood cell count (WBC), blood platelets, absolute neutrophil count (ANC), percentage of neutrophilic (Neut%), absolute lymphocytes count, percentage of lymphocyte (Lymph%), absolute monocyte count (AMC), and percentage of monocyte (Mono%). All these laboratory tests taken during T2DM patients come to the endocrinology centers.

### Statistical analysis

The data was analyzed using SPSS software 26 and R language (4.2.2). Initially, bivariate analysis was used to investigate potential risk variables for diabetic bladder dysfunction, those with normally distributed continuous variables expressed as mean ± standard deviation and analyzed using the independent sample t-test; those with non‐normal distribution expressed as medians (interquartile range) and were analyzed using the Wilcoxon rank‐sum test; and categorical variables were presented as numbers (percentages) and analyzed using the chi-square test. Next, variables that presented a level of significance *p* < 0.05 in the bivariate analysis were included in the multiple logistic regression test. The logistic regression enter method was used to determine independent risk factors of DBD and to construct the prediction model. The prediction model was presented as the model formula. Finally, the receiver operating characteristic (ROC) curve and the area under the ROC curve (AUC) were used to evaluate the prediction value of every risk factor and the DBD predictions model. Internal validation of the model was performed through bootstrap resampling 1000 times to evaluate its reproducibility and check for model overfitting. The model's internal validation results were presented in the AUC and calibration curve. A difference of *p* < 0.05 was considered significant.

### Ethics approval and consent to participate

The studies involving human participants were reviewed and approved by Al Baath University Institutional Review Board Consent Letter NUU–IRB 2023205-S. All procedures were conducted under the ethical principles outlined in the 1964 Declaration of Helsinki and its subsequent revisions. All our methods were carried out under relevant guidelines and regulations. Informed consent was obtained from all the participants and their legal guardian(s). For illiterate participants, informed consent was obtained from legally authorized representatives. We explained the purpose of the study to the patients and their family members before using their data in this study. It was all voluntary; no names were taken, so we provided anonymous data collection.

## Results

### The characteristics of the T2DM patients

This study initially included a total of 233 individuals with T2DM occurring between 2018 and 2023. However, during the screening process, 49 cases were excluded due to various reasons. These reasons encompassed 7 cases with spinal injury,11 cases with Parkinson, 4 cases with stroke, 6 cases with persistent urinary infection, 12 cases with prostate problems, and 9 cases with pelvic organ complications. Finally, 184 individuals with T2DM were included in the statistical analysis, 88 in the DBD group and 96 in the non‐DBD group. Table 1 shows the biochemical and demographic data of patients. Of the 184 T2DM patients, 115 (62.5%) were men, and 69 (37.5%) % were women. The patients' mean age was 61.12 ± 11.48 years, and the duration of T2DM was 6 − 13 years with a median of 9 years. The patient's BMI was 22.48 − 25.81 kg/cm^2^ with a median of 22.72 kg/cm^2^, and the HbA1c was 7.23% − 10.81% with a median of 8.92%.

**Table 1:** Clinical characteristics of the DBD group and the non‐DBD group.

Clinical characteristics	Total (*n* = 184)	DBD group (*n* = 88)	Non‐DBD group (*n* = 96)	*p* Value
Age (years)	61.12 ± 11.48	64.42 ± 11.67	59.62 ± 90.78	0.004
Gender				0.671
Male	115 (62.5%)	56 (63.6%)	59 (61.4%)	
Female	69 (37.5%)	32 (36.4%)	37 (38.6%)	
BMI (kg/cm^2^)	22.72 (22.48 − 25.81)	23.54 (21.73 − 26.52)	23.93 (22.63 − 26.15)	0.592
Duration of T2DM (years)	9.00 (6.00 − 13.00)	10.00 (6.50 − 15.00)	9.00 (5.00 − 13.50)	0.642
Treatment				
Oral drug	105 (56.1%)	46 (52.3%)	59 (63.1%)	0.721
Insulin	48 (25.1%)	27 (30.7%)	21 (20.6%)	
Oral drug + insulin	31 (18.8%)	15 (17.1%)	16 (16.3%)	
Ankle reflexes				0.519
Yes	65 (35.3%)	27 (30.7%)	38 (39.6%)	
No	119 (64.7%)	61 (69.3%)	58 (60.4%)	
Vibration perception				0.629
Yes	107 (58.1%)	62 (70.4%)	45 (46.9%)	
No	77 (41.9%)	26 (29.6%)	51 (53.1%)	
Complications				
DR				0.590
Yes	80 (43.4%)	38 (43.2%)	42 (43.8%)	
No	104 (56.6%)	50 (56.8%)	54 (56.2%)	
PVD				0.739
Yes	94 (51.1%)	42 (47.7%)	52 (54.2%)	
No	92 (49.9%)	46 (54.3%)	44 (45.8%)	
DN				0.427
Yes	53 (25.6%)	32 (28.7%)	30 (22.7%)	
No	131 (74.4%)	65 (71.3%)	66 (77.3%)	
DPN				0.032*
Yes	155 (84.2%)	73 (82.9%)	82 (85.4%)	
No	29 (15.8%)	15 (17.1%)	14 (14.6%)	
Hypertension				0.742
Yes	104 (56.5%)	41 (46.6%)	63 (65.6%)	
No	80 (43.5%)	47 (53.4%)	33 (34.4%)	
Hyperlipaemia				0.631
Yes	115 (62.5%)	42 (47.7%)	73 (76.1%)	
No	69 (37.5%)	46 (52.3%)	23 (23.9%)	
History of stroke				0.582
Yes	19 (10.3%)	8 (9.1%)	11 (11.4%)	
No	165 (89.7%)	80 (90.9.%)	85 (88.6%)	
Coronary heart disease				0.521
Yes	10 (5.4%)	3 (3.4%)	7 (7.3%)	
No	174 (94.6%)	85 (96.6%)	89 (92.7%)	
Laboratory examination				
HbA1c (%)	8.92 (7.23 − 10.81)	8.90 (7.34 − 11.51)	9.72 (8.11 − 11.31)	0.021*
mALB (mg/L)	16.11 (8.41 − 122.17)	24.35 (15.11 − 152.21)	10.55 (5.32 − 98.24)	0.008*
TG (mmol/L)	1.27 (0.71 − 2.04)	1.24(0.62 − 1.73)	1.38 (0.83 − 3.47)	0.361
TC (mmol/L)	3.67 (2.19 − 4.47)	3.94 (3.41 − 4.82)	3.82 (2.91 − 4.33)	0.422
HDL‐C (mmol/L)	1.05 (0.77 − 1.31)	1.09 (0.81 − 1.41)	1.04 (0.84 − 1.75)	0.437
LDL‐C (mmol/L)	2.39 (1.31 − 2.54)	2.41 (1.53 − 2.92)	2.11 (1.21 − 2.84)	0.462
Urea (mmol/L)	5.01 (3.12 − 6.48)	5.24 (3.47 − 6.81)	5.03 (3.02 − 6.37)	0.371
SCr (μmol/L)	72.41 (55.20 − 91.64)	75.32 (56.40 − 98.73)	72.50 (55.92 − 85.11)	0.132
UA (μmol/L)	325.13 (248.40 − 377.23)	327.41 (249.09 − 391.21)	324.08 (254.21 − 373.14)	0.267
RBC (× 10^12/L)	3.27 (3.01 − 3.95)	3.32 (2.91 − 3.85)	3.47 (3.07 − 4.19)	0.048*
WBC (× 10^9/L)	6.21 (5.22 − 7.58)	6.31 (5.37 − 8.13)	6.19 (5.32 − 7.46)	0.008*
PLTs (× 10^9/L)	214 (162.11 − 254.72)	215.00 (164.75 − 251.00)	220.06 (163.55 − 262.36)	0.489
ANC (× 10^9/L)	3.82 (3.11 − 5.42)	4.21 (3.01 − 7.01)	3.51 (2.74 − 5.81)	0.045*
Neut% (%)	56.42 (51.34 − 65.73)	58.21 (52.81 − 74.11)	55.84 (51.78 − 64.93)	0.032*
ALC (× 10^9/L)	1.74 (1.28 − 2.23)	1.72 (1.26 − 2.18)	1.77 (1.32 − 2.41)	0.471
Lymph% (%)	22.49 (18.47 − 31.28)	23.10 (15.11 − 31.16)	24.84 (20.24 − 34.18)	0.046*
AMC (× 10^9/L)	0.41 (0.33 − 0.49)	0.37 (0.29 − 0.48)	0.44 (0.34 − 0.53)	0.417
Mono% (%)	5.89 (5.24 − 7.04)	5.42 (5.11 − 6.41)	6.49 (5.61 − 7.12)	0.009*

Note: Data are n (%), mean ± SD or medians (interquartile range); p‐value for differences between groups were obtained by χ2 test, ANOVA, or the Wilcoxon rank‐sum test. Abbreviations: ALC, absolute lymphocytes count; AMC, absolute monocyte count; ANC, absolute neutrophil count; ANOVA, analysis of variance; BMI, body mass index; DBD, diabetic bladder dysfunction; DN, diabetic nephropathy; DPN, diabetic peripheral neuropathy; DR, diabetic retinopathy; HbA1c, glycated hemoglobin; HDL‐C, high‐density lipoprotein cholesterol; LDL‐C, low‐density lipoprotein‐cholesterol; Lymph%, percentage of lymphocyte; mALB, urinary microalbuminuria; Mono%, percentage of monocyte; Neut%, percentage of neutrophilic; PLTs, platelets count test PVD, diabetic peripheral vascular disease; RBC, red blood cell count; SCr, serum creatinine; TC, total cholesterol; TG, triglyceride; UA, uric acid; WBC, white blood cell count; T2DM, type 2 diabetes mellitus. *p < 0.05.

### Risk factors associated with DBD in bivariate analyses

The correlations between each variable with DBD were shown in Table 1. Our bivariate analysis revealed that age, DPN, HbA1c, mALB, RBC, WBC, ANC, Mono, Neut%, and Lymph% were all statistically significant differences between the two groups (*p* < 0.05). Even though the duration of T2DM and renal function tests (UREA, SCr) were not statistically significant between the two groups (p > 0.05), the mean value of T2DM duration in the DBD group was greater than that in the non-DBD group (10 vs. 9 years), the mean value of renal function tests (UREA, SCr) was greater than that in the non-DBD group (5.24 vs. 5.03 mmol/L, 75.32 vs. 72.50 μmol/L).

### Independent risk factors screening and model construction

All variables with a significant p-value in the bivariate analysis were entered into the multiple logistic regression analysis (Table 2). Our results presented that age (OR [95% CI]: 0.981 [0.614 − 1.337]), *p* = 0.007; DPN (OR [95% CI]: 1.421 [1.027 − 3.308]), *p* = 0.031; HbA1c (OR [95% CI]: 0.942 [0.821 − 1.141]), *p* = 0.042; and Mono (OR [95% CI]: 1.109 [0.815 − 1.258]), *p* = 0.031 were independent risk factors. The final formula for the prediction model was Logit (p) =  − 3.728 + 0.024 age + 0.462 DPN + 0.087 HbA1c + 0.051 Mono%.

**Table 2:** Logistic regression analysis of bladder dysfunction in patients with type 2 diabetes mellitus.

Factors	β‐coefficient	Wald‐test	*p* Value	OR (95% CI)
Age	0.024	11.315	0.007*	0.981 (0.614 − 1.337)
DPN	0.462	4.814	0.031*	1.421 (1.027 − 3.308)
HbA1c	0.087	3.919	0.042*	0.924 (0.821 − 1.141)
mALB	0.041	1.238	0.617	0.827 (0.619 − 0.962)
RBC	0.201	0.310	0.517	0.926 (0.432 − 1.381)
WBC	0.039	0.052	0.503	0.872 (0.631 − 1.107)
ANC	0.051	4.371	0.055	1.031 (0.537 − 1.274)
Mono%	0.062	3.247	0.031*	1.109 (0.815 − 1.258)
Neut% (%)	0.082	7.213	0.085	1.023 (1.043 − 1.372)
Lymph% (%)	0.053	5.386	0.067	1.142 (0.783 − 1.193)
Constant	-3.728	6.735	< 0.004*	1.174 (1.089 − 1.313)

Abbreviations: ANC, absolute neutrophil count; DPN, diabetic peripheral neuropathy; HbA1c, glycated hemoglobin; mALB, urinary microalbuminuria; Mono%, percentage of monocyte; OR (95% CI), odds ratios (95% confidence intervals); RBC, red blood cell count; WBC, white blood cell count; ‐, means null value. *p < 0.05.

### Predictive accuracy of independent risk factors and the prediction model

**Figure 1:**
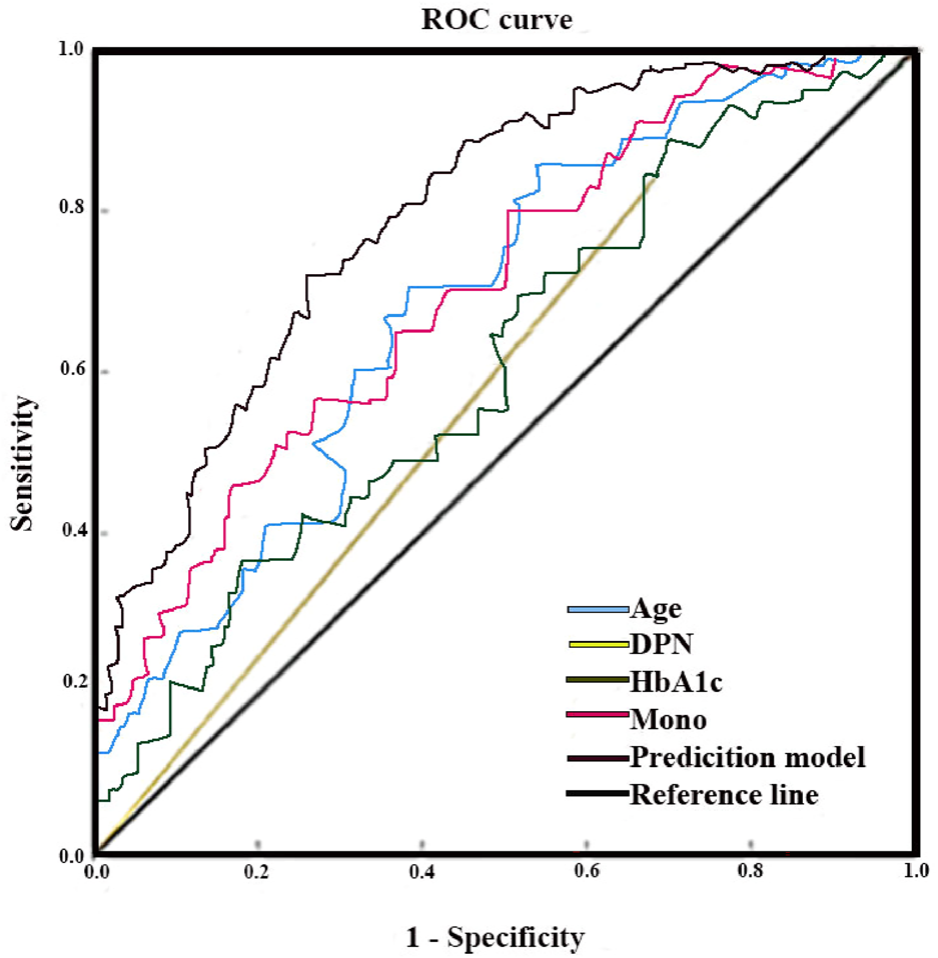
ROC curve of independent risk factors and the prediction model with bladder dysfunction in type 2 diabetes mellitus. Mono, percentage of monocytes; DPN, diabetic peripheral neuropathy; HbA1c, glycated hemoglobin; ROC, receiver operating characteristic.

**Figure 2:**
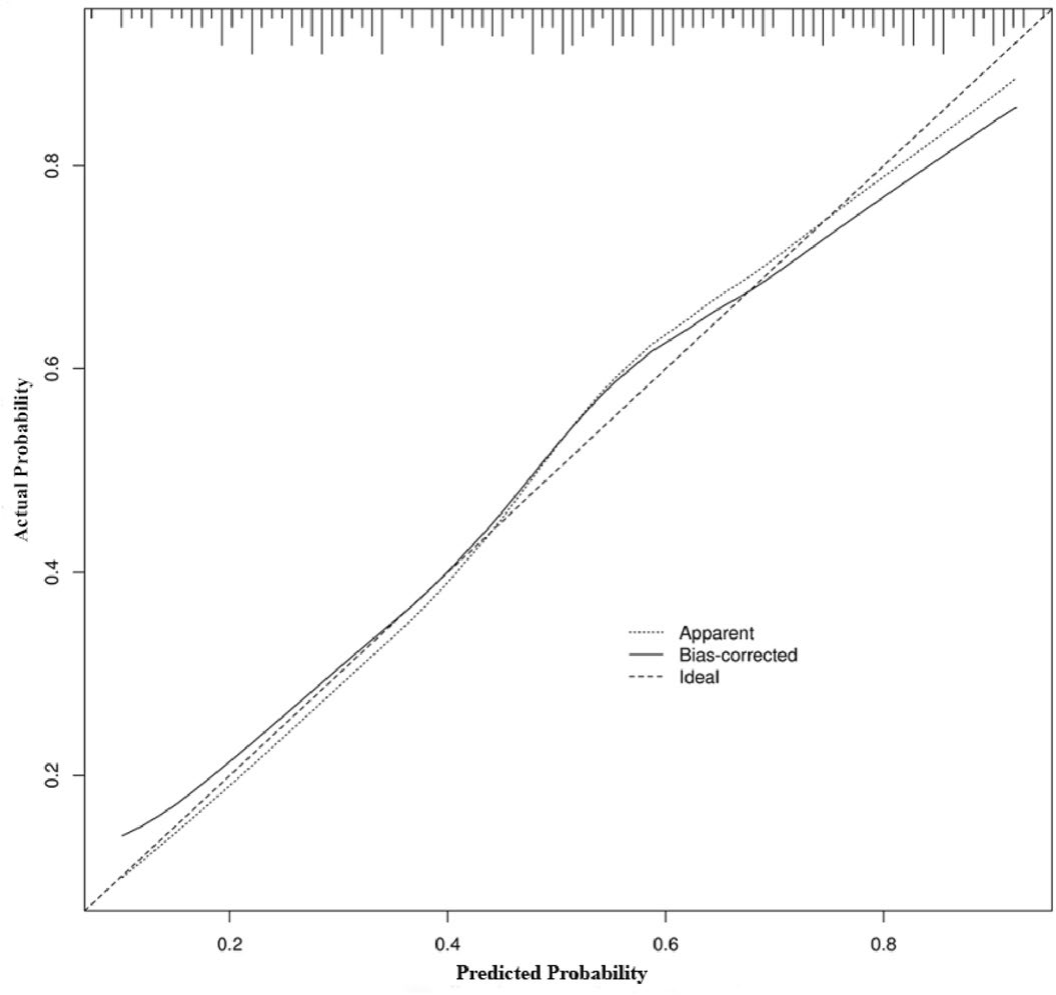
Calibration curve for the internal validation of the prediction model.

The ROC curve analysis revealed that the AUC of age, DPN, HbA1c, and Mono were 0.703, 0.541, and 0.613, and 0.836, respectively; the sensitivity was 81.4%, 70.6%, 84.5%, and 87.4%, respectively; the specificity was 32.4%, 41.3%, and 32.7%, and 48.7%, respectively. The ROC curve analysis of the prediction model showed that AUC = 0.684 (95% CI: 0.601 − 0.749), with the sensitivity of 76.3% and specificity of 34.6%, respectively (Fig. 1 and Table 3). The model was internally validated by boostrap resampling 1000 times, AUC = 0.675 (95% CI: 0.611 − 0.807), and the calibration curve of the model was close to the ideal diagonal line (Fig. 2).

**Table 3:** ROC curve analysis of independent risk factors and the prediction model with bladder dysfunction in type 2 diabetes mellitus.

	AUC	95% CI	Sensitivity (%)	Specificity (%)	Cutoff value	*p* Value
Age	0.703	(0.652 − 0.739)	81.4	32.4	–	0.008
DPN	0.541	(0.483 − 0.648)	70.6	41.3	–	0.036
HbA1c	0.613	(0.524 − 0.671)	84.5	32.7	6.46	< 0.001
Mono%	0.836	(0.741 − 0.867)	87.4	48.7	4.62	< 0.001
Prediction model	0.684	(0.601 − 0.749)	76.3	34.6	46%	0.016

Note: Cutoff value is the critical value, the > cutoff value is determined to be positive, and the < cutoff value is judged to be negative; ‐ means null value. Abbreviations: Mono%, percentage of monocyte, area under the receiver operating characteristic curve; DPN, diabetic peripheral neuropathy; HbA1c, glycated hemoglobin; ROC, receiver operating characteristic; 95% CI, 95% confidence intervals.

## Discussion

In the current study, four risk variables for the development of diabetic bladder dysfunction (DBD) were identified: age, diabetic peripheral neuropathy (DPN), glycated hemoglobin (HbA1c), andpercentage of monocytes (Mono%); the AUC was used to assess the DBD prediction model and the predictive value of each risk factors. When AUC is greater than 0.5, the prediction of DBD is meaningful; when AUC is greater than 0.8, the prediction is good^17^.

We found that individual risk factors had limited predictive power for the occurrence of DBD, but they had some predictive value when they combined to predict the occurrence of DBD (AUC = 0.684). Internal validation of the model was performed (AUC = 0.675), which indicates meaningful accuracy. In addition, this study demonstrated that individuals with T2DM who were above the age of 59.6 years were more likely to develop DBD. This result is in line with prior research^18,19^. Age has been identified as an independent factor associated with urinary problems in TIDM^8^ and DBD in T2DM^19^. The incidence of T2DM increases with age^20^, and the aging process contributes to β cell dysfunction and high blood glucose levels^21^. Persistent elevated blood glucose levels can lead to oxidative stress and damage to nerves and muscles, including those involved in bladder function. Previous studies have found that the duration of diabetes increased the prevalence of DBD, among patients with a DM duration of 8 to 9 years being more likely to develop DBD^22^. Although the duration of T2DM in this study did not show statistical significance as a risk factor for DBD, the mean duration of T2DM in the DBD group was much longer compared to the non-DBD group. Therefore, in clinical practice, we should pay attention to screening patients with an age > 59.6 years and a long duration of diabetes to prevent and reduce the occurrence of DBD.

This study found that HbA1c was an independent risk factor for DBD. The HbA1c test is the gold standard method for determining average blood glucose levels over the past 120 days^23^ and for evaluating glycemic management, and it is associated with the development and progress of DM and its consequences^24–26^. Previous research has also shown an association between HbA1c and urinary complications in T2DM^8^. A study conducted by Efstathios Papaefstathiou et al. among individuals with T2DM demonstrated a 2.5-fold greater risk of lower urinary tract symptoms for each unit rising in HbA1c^27^. Another study conducted by Aih-Fung Chiu et al. found a significant association between high HbA1c levels and overactive bladder/urgency^28^. Notably, one study found that women with HbA1c levels higher than 8.4 were at a higher risk of urinary complications^29^. As a result, patients with poor blood glucose control should be monitored early and addressed appropriately.

Furthermore, DBD is considered to be a subset of diabetic autonomic neuropathy. A study summarized that the relationship between diabetic autonomic neuropathy and the genitourinary tract revealed that diabetic autonomic neuropathy was associated with developing symptoms related to the urinary tract^30^. Diabetic neuropathy is a common consequence of diabetes, and there is a high coexistence of diabetic autonomic neuropathy and DPN^31^. In our study, individuals with DPN had 1.5 times higher odds of developing DBD compared to those without DPN. However, the predictive value of DPN for the occurrence of DBD was limited; this may be attributed to the inability to measure the degree of severity of DPN in this study. Another study performed among individuals with T2DM demonstrated a significant association between bladder neuropathy and somatic and arterial peripheral neuropathy^32^. These outcomes suggest that somatic peripheral neuropathy and bladder dysfunction may share common underlying mechanisms^33^. In summary, in clinical practice, we should not only pay attention to observing patients with diabetic autonomic neuropathy but also observe patients with DPN, all of whom are at risk of DBD.

Blood routine is the most essential clinical blood test project, a necessary basis for doctors to diagnose diseases. This study showed that the Mono% was an independent risk factor for DBD. In addition, ANC% was statistically significant in the bivariate logistic regression analysis. Oxidative stress has been implicated in diabetes-related complications, and numerous studies have demonstrated the association between oxidative stress and these complications^34^. Phagocytic immune cells produce reactive oxidative species (ROS) and free radicals when activated. Excessive ROS and free radicals production leads to oxidative stress^35,36^. In diabetic patients, the oxidative stress response can result in morphological and functional alternations in the urothelium, resulting in the development of DBD^37^. Monocytes, as phagocytic immune cells, serve as indicators of the oxidative stress response^38^. This study revealed that the Mono% values were identified as an independent risk factor for diabetic bladder dysfunction (DBD). The current study showed that a high level of Mono was one of the factors that led to the development of DBD. Further research can explore the relationship between regular blood values and DBD in more detail, shedding light on potential preventive measures that can be implemented based on these findings.

## Conclusion

In conclusion, the prediction model developed in this study using the factors of age, diabetic peripheral neuropathy (DPN), HbA1c, and percentage of monocytes (Mono%) showed good clinical value in screening patients at risk of diabetic bladder dysfunction (DBD). Timely treatment and care can be provided to patients to avoid or minimize the development of DBD. However, it is important to note that this study had some limitations. It was a retrospective study with a small sample size, which may introduce information bias and limit the generalizability of the findings. Moreover, the study focused on collecting objective data and did not consider other factors, such as lifestyle and psychological aspects, that may influence the development of DBD. In addition, this study did not classify the patients with BDD into three groups: severe, moderate, and mild since this classification allows for a more focused and targeted analysis of outcomes, enabling more profound insights into the pathophysiology, risk factors, treatment responses, and long-term outcomes associated with each severity group.

Future research should involve larger sample sizes and longitudinal study designs to gain a more comprehensive understanding of DBD and its predictors. Additionally, it would be valuable to explore the impact of other factors, including lifestyle factors and psychological aspects, on the development of DBD and classify the patients with BDD into three groups: severe, moderate, and mild; this would provide a more holistic perspective on the condition and aid in developing comprehensive prevention and management strategies.

## Data Availability

The datasets generated and/or analyzed during the current study are not publicly available due [to maintain the privacy of the patients participating in the study] but are available from the corresponding author on reasonable request.
